# Association between serum 25-hydroxyvitamin D levels and cardiometabolic index among overweight/obese U.S. adults: A cross-sectional study

**DOI:** 10.1097/MD.0000000000047304

**Published:** 2026-01-16

**Authors:** Jingwen Zhang, Yuxing Tai, Sixian Wang, Mingrui Chu, Rongsheng Jiang, Zhengri Cong, Qifan Guan, Mingjun Liu

**Affiliations:** a Department of Acupuncture and Tuina, Changchun University of Traditional Chinese Medicine, Changchun, Jilin, China; b Department of Acupuncture and Tuina, Jiangxi University of Traditional Chinese Medicine, Nanchang, Jiangxi, China.

**Keywords:** 25-hydroxyvitamin D, cardiometabolic index, cross-sectional study, NHANES, obese, overweight, vitamin D

## Abstract

Obesity is a major driver of cardiovascular disease (CVD) risk, with the cardiometabolic index (CMI) serving as a novel indicator. While vitamin D’s potential protective role against CVD is recognized, its precise dose-response relationship with CMI in high-risk overweight/obese individuals remains unclear. This study aimed to systematically evaluate the association between serum 25-hydroxyvitamin D (25(OH)D) levels and CMI in overweight/obese U.S. adults and characterize its dose-response pattern. A cross-sectional analysis was conducted using data from the National Health and Nutrition Examination Survey 2009 to 2018, including 6597 overweight/obese participants aged 18 to 64 years. Serum 25(OH)D levels were categorized into 3 groups: sufficient (≥75 nmol/L), insufficient (50–74 nmol/L), and deficient (<50 nmol/L). CMI was calculated as (triglycerides/high-density lipoprotein cholesterol) × waist-to-height ratio. Multivariable linear regression models were employed to assess the association of 25(OH)D with CMI. Restricted cubic splines and likelihood ratio tests for piecewise regression were used to rigorously test for nonlinearity. Subgroup analyses and interaction tests were performed to evaluate the heterogeneity of the association across different populations. After comprehensive adjustment, serum 25(OH)D levels were significantly and independently associated with CMI in a negative linear manner (β = −0.09, 95% CI: −0.14 to −0.05, *P* <.001). Compared to the sufficient 25(OH)D group, the deficient group had a significantly higher CMI (β = 0.19, 95% CI: 0.10 to 0.28, *P* <.001). Both restricted cubic splines (P for nonlinearity = 0.389) and LRT (*P* = .346) did not support a nonlinear association. Notably, this negative association was significantly stronger in participants with diabetes (P for interaction = 0.038). The effect size (β = −0.21, 95% CI: −0.37 to −0.05) was more than 3 times that of the nondiabetic population (β = −0.06, 95% CI: −0.11 to −0.02) (P for interaction = 0.038). This study found that in overweight/obese U.S. adults, higher serum 25(OH)D levels are linearly associated with better cardiometabolic health (lower CMI), with this association being more pronounced in individuals with diabetes. These findings suggest that improving vitamin D status may be a potential public health strategy for mitigating cardiometabolic risk in the overweight/obese population.

## 1. Introduction

The escalating global prevalence of obesity has become one of the most pressing public health challenges of the 21st century. It is projected that by 2030, over 50% of the global adult population will be affected, with nearly 3 billion adults facing overweight or obesity.^[1]^ This condition, characterized by excessive fat accumulation, is not merely an independent disease state but also fosters a unique pathological environment of chronic low-grade inflammation, insulin resistance, and lipid metabolism disorders. This environment directly contributes to a series of metabolic complications, such as hypertension and atherosclerosis, ultimately leading to severe cardiovascular events like coronary heart disease and stroke, which are major contributors to global morbidity and mortality.^[2,3]^ For adults, obesity not only impairs quality of life and labor productivity but also imposes an increasing burden on household finances and societal healthcare systems through its associated cardiovascular complications, profoundly impacting socioeconomic development.^[4,5]^ Therefore, accurately identifying and intervening in the cardiometabolic risk factors of this high-risk population is of critical public health significance for curbing the trend of cardiovascular disease.

Against this backdrop, identifying a biomarker that can accurately reflect the overall degree of cardiometabolic derangement in individuals with obesity is paramount. Traditional single indicators, such as isolated lipid or glucose levels, often fail to capture the full picture. The cardiometabolic index (CMI), a novel composite indicator, is well-suited for assessing metabolic risk in the population with obesity and has been significantly associated with hypertension, diabetes, hyperuricemia, and cardiovascular disease risk in numerous studies.^[6–9]^ It ingeniously integrates 2 core pathophysiological aspects of obesity-related metabolic disturbances: insulin resistance-driven dyslipidemia (represented by the triglycerides to high-density lipoprotein cholesterol ratio) and visceral fat accumulation-driven central obesity (represented by the waist-to-height ratio).^[10,11]^ This multidimensional integration allows CMI to capture cardiometabolic risk more comprehensively and sensitively than single anthropometric measures.^[12]^

Vitamin D, as a functional vitamin, has physiological roles that extend far beyond its classical function in maintaining bone health. The vitamin D receptor (VDR) is widely expressed in key tissues involved in glucose metabolism and blood pressure regulation, such as vascular endothelial cells, suggesting that vitamin D may exert important protective effects on cardiometabolic health through complex mechanisms, including influencing insulin signaling pathways and improving endothelial function.^[13,14]^ Several epidemiological studies have begun to reveal a significant association between vitamin D deficiency or insufficiency and an increased risk for the development and progression of cardiometabolic diseases.^[15–18]^ However, substantial evidence indicates that individuals with increased adiposity commonly present with low serum 25-hydroxyvitamin D (25(OH)D) levels due to unique dietary patterns, lifestyle factors, and sequestration in adipose tissue.^[19–22]^ This phenomenon may create a vicious cycle: obesity leads to functional vitamin D deficiency, which in turn may exacerbate obesity-related metabolic disorders by intensifying inflammation and impairing insulin signaling pathways.^[23–25]^ To date, most studies have only explored the association between vitamin D and individual components of cardiometabolic risk, failing to unveil its integrated impact on overall cardiometabolic health within the specific pathophysiological state of obesity.^[22,26,27]^

Therefore, elucidating the relationship between serum 25(OH)D levels and CMI, particularly in this “dual-high” population (high prevalence of both vitamin D deficiency and cardiometabolic disease), is of significant clinical and public health importance. This study aims to utilize the large-scale, nationally representative data from the U.S. National Health and Nutrition Examination Survey (NHANES) to systematically evaluate the association between serum 25(OH)D and CMI in overweight/obese U.S. adults. By employing advanced statistical models, we will rigorously characterize the dose-response pattern of this association, with the goal of providing new, evidence-based insights for the management of cardiometabolic risk in this critical population.

## 2. Materials and methods

### 2.1. Study population

**Figure 1.**
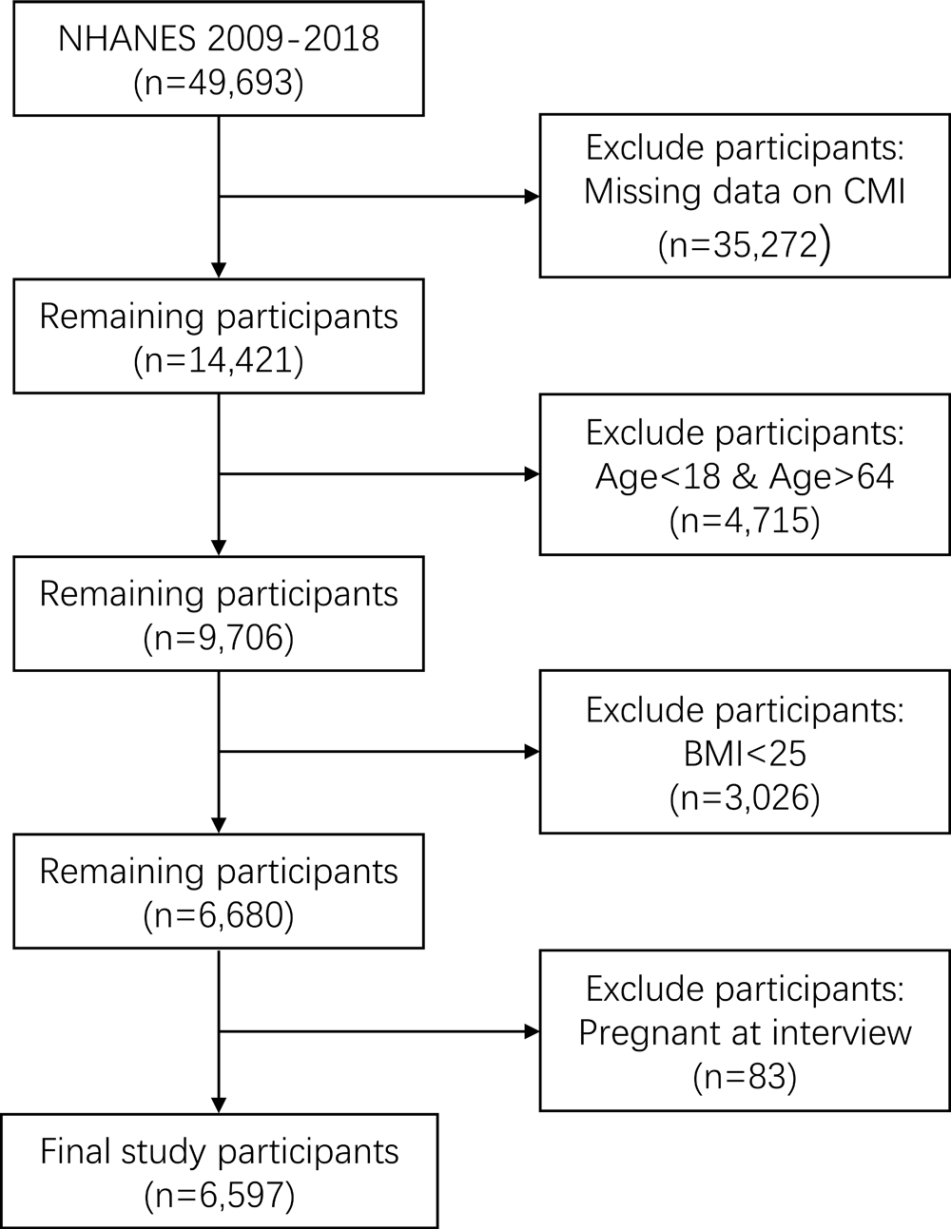
Participants screening flowchart. BMI = body mass index, CMI = cardiometabolic index, NHANES = National Health and Nutrition Examination Survey.

NHANES is a nationwide health monitoring program led by the U.S. Centers for Disease Control and Prevention, designed to comprehensively assess the health and nutritional status of the U.S. population through questionnaires, physical examinations, and laboratory tests. In this study, we utilized data from the 2009 to 2018 NHANES cycles. The NHANES protocols were approved by the National Center for Health Statistics Research Ethics Review Board. As this study involved secondary analysis of publicly available, de-identified data, it was deemed exempt from further institutional review board approval by our institution. From an initial sample of 49,693 participants between 2009 and 2018, we excluded those with missing data for CMI calculation (n = 35,272), those outside the age range of 18 to 64 years (n = 4715), those with a Body Mass Index (BMI) <25 kg/m² (n = 3026), and those who were pregnant at the time of the interview (n = 83). Ultimately, 6597 participants were included in the final analysis (Fig. 1).

### 2.2. Exposure variable

The exposure variable in this study was the total serum 25-hydroxyvitamin D (25(OH)D) level, calculated as the sum of 25-hydroxyvitamin D3 (25(OH)D3) and 25-hydroxyvitamin D2 (25(OH)D2), expressed in nmol/L. The measurements were conducted by the CDC using high-performance liquid chromatography-tandem mass spectrometry. The entire measurement process strictly adhered to quality control and quality assurance protocols to ensure data reliability. Detailed laboratory methodologies and quality control procedures can be referenced in the NHANES laboratory methods files for the specific survey cycles.

For ease of analysis, this study categorized serum 25(OH)D levels according to the 2011 clinical practice guidelines published by the Endocrine Society^[28]^: Q1, sufficient (≥75 nmol/L); Q2, insufficient (50–74 nmol/L); and Q3, deficient (<50 nmol/L). Q1 was used as the reference group in subsequent analyses.

### 2.3. Outcome variable

The outcome variable of this study was the Cardiometabolic Index (CMI), which is calculated using 4 physiological indicators: serum triglycerides (TG), high-density lipoprotein cholesterol (HDL-C), waist circumference (WC), and height. TG was measured using a timed-endpoint enzymatic method, while HDL-C was measured via a direct method; both were reported in mmol/L. WC and height were measured by trained technicians using a measuring tape at the level of the iliac crest and a stadiometer in a standing position, respectively, with units in cm. Prior to calculating CMI, the waist-to-height ratio (WHtR) was first computed (WC/height). The formula for CMI is as follows:


$$\begin{array}{l} CMI=\frac{\;TG\;\;\;\left(mmol/L\right)}{\;HDL-C\;\;\;\left(mmol/L\right)}\times WHtR \end{array}$$

### 2.4. Covariate

The covariates included in this study are recognized in the literature as potent predictors of cardiometabolic disease risk.^[9,27,29]^ Detailed measurement protocols and data collection methods are publicly available at: https://wwwn.cdc.gov/nchs/nhanes/analyticguidelines.aspx. These covariates include: sociodemographic variables (age, gender, race, poverty-income ratio (PIR)); anthropometric variables (weight, height, BMI, WC, and WHtR); lifestyle variables (smoking status, alcohol consumption, and moderate-intensity physical activity); and personal health variables (history of sleep disorders, diabetes, and hypertension). Gender was classified as male or female. Race/ethnicity was categorized as Mexican American, Non-Hispanic Black, Non-Hispanic White, Other Hispanic, and Other Race (including Multi-Racial). PIR was categorized as low (<1.3), middle (1.3–3.5), and high (>3.5). BMI was classified according to WHO standards as overweight (25–30 kg/m²) and obese (≥30 kg/m²). Smoking status was defined as having smoked ≥ 100 cigarettes/lifetime versus <100 cigarettes/lifetime. Drinking status was defined as having ≥ 12 alcoholic drinks/lifetime versus <12 alcoholic drinks/lifetime. Moderate-intensity physical activity was categorized as ≥ 10 minutes/week versus <10 minutes/week (Table 1).

**Table 1:** Classification of covariates.

Covariates	Classification
Gender	Male; Female
Race	Mexican American; Non-Hispanic Black; Non-Hispanic White; Other Hispanic; Other Race – Including Multi-Racial
Poverty-income ratio	Low income(<1.3); Middle income(1.3–3.5);High income(≥3.5)
BMI	Overweight(25–30); Obesity(≥30)
Smoking Status	≥100 cigarettes/lifetime; <100 cigarettes/lifetime
Drinking Status	≥12 alcohol drinks/lifetime; <12 alcohol drinks/lifetime
Moderate-intensity sports	≥10 minutes/week; <10 minutes/week
Sleep disorders	Yes; No
Hypertension	Yes; No
Diabetes	Yes; No

BMI = body mass index

### 2.5. Statistical analysis

All analyses in this study strictly adhered to the complex sampling design guidelines of NHANES to ensure the accuracy and generalizability of the results. Continuous variables are presented as mean ± standard deviation (Mean ± SD) or median (interquartile range, IQR), while categorical variables are described using frequencies (n) and percentages (%). ANOVA, Kruskal-Wallis H test, chi-square test, or Fisher exact test were used to compare differences between groups. To analyze the association between serum 25(OH)D levels and CMI, we constructed 3 linear regression models with 25(OH)D entered as both a continuous and a categorical variable: Model 1 (unadjusted), Model 2 (adjusted for age, gender, race, and PIR), and Model 3 (further adjusted for BMI, smoking status, drinking status, physical activity, sleep disorders, diabetes, and hypertension, in addition to variables in Model 2). Regression coefficients (β) and their 95% confidence intervals are reported for all models. Restricted cubic splines (RCS) were used to model the dose-response relationship to explore potential nonlinearity. A 2-piecewise linear regression model was employed to identify a potential threshold effect of serum 25(OH)D on CMI. To assess the heterogeneity of the association, we conducted subgroup analyses stratified by gender, race, PIR, BMI, smoking status, physical activity, sleep disorders, diabetes, and hypertension. Furthermore, to test the robustness of our findings, we conducted a sensitivity analysis by repeating the multivariable linear regression (Model 3) exclusively in the subgroup of participants with obesity (BMI ≥30 kg/m²).In all analyses, a 2-sided *P*-value <.05 was considered statistically significant.

All analyses were performed using R Statistical Software (Version 4.2.2, http://www.R-project.org, The R Foundation) and Free Statistics analysis platform (Version 2.0, Beijing, China).

## 3. Results

### 3.1. Baseline characteristics of study population

This study ultimately included 6597 overweight/obese adult participants who met the inclusion and exclusion criteria. The mean age was 42.8 years (SD = 13.3), with 3265 males (49.5%) and 3332 females (50.5%). Significant statistical differences were observed in the baseline characteristics among the different serum 25(OH)D level groups (all *P*-values <.05). Compared to individuals with lower serum 25(OH)D levels, those in the sufficient 25(OH)D group were older, more likely to be Non-Hispanic White, had higher incomes, and adopted healthier lifestyle behaviors (e.g., less smoking, more physical activity). Conversely, participants in the deficient 25(OH)D group had significantly higher mean weight, BMI, and WC. The median CMI in the deficient 25(OH)D group was slightly higher than that in the sufficient group (Table 2).

**Table 2:** Baseline characteristics of the study population.

Variables	Total	Q1	Q2	Q3	*P*-value
	n = 6597	n = 1522	n = 2590	n = 2485	
Age, Mean ± SD	42.8 ± 13.3	47.1 ± 12.6	42.6 ± 13.1	40.5 ± 13.4	<.001
Gender, n (%)					
Male	3265 (49.5)	715 (47)	1382 (53.4)	1168 (47)	<.001
Female	3332 (50.5)	807 (53)	1208 (46.6)	1317 (53)	
Race, n (%)					
Mexican American	1295 (19.6)	141 (9.3)	550 (21.2)	604 (24.3)	<.001
Non-Hispanic Black	799 (12.1)	146 (9.6)	416 (16.1)	237 (9.5)	
Non-Hispanic White	2300 (34.9)	900 (59.1)	970 (37.5)	430 (17.3)	
Other Hispanic	1525 (23.1)	193 (12.7)	404 (15.6)	928 (37.3)	
Other Race – Including Multi-Racial	678 (10.3)	142 (9.3)	250 (9.7)	286 (11.5)	
PIR, n (%)					<.001
Low income(<1.3)	2331 (35.3)	457 (30)	938 (36.2)	936 (37.7)	
Middle income(1.3–3.5)	2435 (36.9)	495 (32.5)	943 (36.4)	997 (40.1)	
High income(≥3.5)	1831 (27.8)	570 (37.5)	709 (27.4)	552 (22.2)	
Weight Mean ± SD	91.1 ± 20.3	88.8 ± 18.4	90.0 ± 19.8	93.6 ± 21.6	<.001
Height, Mean ± SD	167.8 ± 10.1	168.4 ± 10.2	168.1 ± 10.2	167.3 ± 9.9	.001
BMI, n (%)					<.001
Overweight(25–30)	2927 (44.4)	793 (52.1)	1205 (46.5)	929 (37.4)	
Obesity(≥30)	3670 (55.6)	729 (47.9)	1385 (53.5)	1556 (62.6)	
WC, Mean ± SD	105.6 ± 14.6	104.1 ± 13.7	104.7 ± 14.2	107.4 ± 15.5	<.001
WHtR, Mean ± SD	0.6 ± 0.1	0.6 ± 0.1	0.6 ± 0.1	0.6 ± 0.1	<.001
TG, Median (IQR)	1.5 ± 1.5	1.5 ± 1.3	1.6 ± 1.5	1.5 ± 1.6	.033
HDL-C, Mean ± SD	1.3 ± 0.4	1.4 ± 0.4	1.3 ± 0.4	1.3 ± 0.3	<.001
CMI, Median (IQR)	0.6 (0.4, 1.0)	0.6 (0.3, 1.0)	0.6 (0.4, 1.1)	0.6 (0.3, 1.0)	<.001
Smoking status, n (%)					
≥100 cigarettes/lifetime	2745 (41.6)	686 (45.1)	1105 (42.7)	954 (38.4)	<.001
<100 cigarettes/lifetime	3852 (58.4)	836 (54.9)	1485 (57.3)	1531 (61.6)	
Drinking status, n (%)					
≥12 alcohol drinks/lifetime	3615 (54.8)	884 (58.1)	1430 (55.2)	1301 (52.4)	.002
<12 alcohol drinks/lifetime	2982 (45.2)	638 (41.9)	1160 (44.8)	1184 (47.6)	
Moderate-intensity sports, n(%)					
≥10 minutes/week	2663 (40.4)	715 (47)	1045 (40.3)	903 (36.3)	<.001
<10 minutes/week	3934 (59.6)	807 (53)	1545 (59.7)	1582 (63.7)	
Sleep disorders, n (%)					
Yes	1758 (26.6)	524 (34.4)	635 (24.5)	599 (24.1)	<.001
No	4839 (73.4)	998 (65.6)	1955 (75.5)	1886 (75.9)	
Diabetes, n (%)					
Yes	790 (12.0)	219 (14.4)	295 (11.4)	276 (11.1)	.004
No	5807 (88.0)	1303 (85.6)	2295 (88.6)	2209 (88.9)	
Hypertension, n (%)					
Yes	2207 (33.5)	636 (41.8)	768 (29.7)	803 (32.3)	<.001
No	4390 (66.5)	886 (58.2)	1822 (70.3)	1682 (67.7)	

BMI = body mass index, CMI = cardiometabolic index, HDL-C = high-density lipoprotein cholesterol, PIR = poverty-income ratio, TG = triglycerides, WC = waist circumference, WHtR = waist-to-height ratio.

### 3.2. The association between 25(OH)D and CMI

Multiple linear regression analysis was used to investigate the association between serum 25(OH)D levels and CMI (Table 3). When serum 25(OH)D was treated as a continuous variable in the unadjusted Model 1, no significant association with CMI was observed (β = −0.03, 95% CI: −0.07 to 0.01, *P* = .201). However, after adjusting for age, gender, race, and PIR in Model 2, a significant negative association emerged (β = −0.12, 95% CI: −0.16 to −0.08, *P* <.001). This negative association remained significant after full adjustment in Model 3 (β = −0.09, 95% CI: −0.14 to −0.05, *P* <.001). When serum 25(OH)D level was modeled as a categorical variable with Q1 as the reference, the CMI of the Q2 group was significantly higher than the Q1 group in Model 1 (β = 0.13, 95%CI: 0.04–0.21, *P* = .003), while the difference between the Q3 and Q1 groups was not significant (β = 0.07, 95%CI: −0.01–0.16, *P* = .094), and the test for trend was not significant (*P* = .201). In the fully adjusted Model 3, the CMI was significantly higher in both the Q2 group (β = 0.14, 95%CI: 0.05–0.22, *P* = .001) and the Q3 group (β = 0.19, 95%CI: 0.10–0.28, *P* <.001) compared to the Q1 group, with a significant trend observed (*P* for trend <.001). Compared to individuals with sufficient serum 25(OH)D, those with insufficient or deficient levels have a higher CMI.

**Table 3:** Multiple linear regression analysis of relationship of serum 25-hydroxyvitamin D with cardiometabolic index.

Variable	Model 1		Model 2		Model 3	
	Β (95% Cl)	*P*-value	Β (95% Cl)	*P*-value	Β (95% Cl)	*P*-value
25(OH)D	-0.03 (-0.07–0.01)	.201	−0.12 (−0.16 to −0.08)	<.001	−0.09 (-0.14 to −0.05)	<.001
25(OH)D categorical						
Q1(≥75)	Reference		Reference		Reference	
Q2(50–75)	0.13 (0.04–0.21)	.003	0.15 (0.07–0.24)	<.001	0.14 (0.05–0.22)	.001
Q3(<50)	0.07 (−0.01 to 0.16)	.094	0.25 (0.16–0.33)	<.001	0.19 (0.1–0.28)	<.001
*P* for trend	0.03 (−0.01 to 0.07)	.201	0.12 (0.08–0.16)	<.001	0.09 (0.05–0.14)	<.001

Model 1 was unadjusted.

Model 2 was adjusted for Model 1 + age, gender, race, poverty-income ratio.

Model 3 was adjusted for Model 2 + BMI, smoking, drinking, activity, sleep disorders, diabetes and hypertension.

β = coefficient, BMI = body mass index, CI = confidence interval.

### 3.3. Dose-response relationship analysis between 25(OH)D and CMI

**Figure 2.**
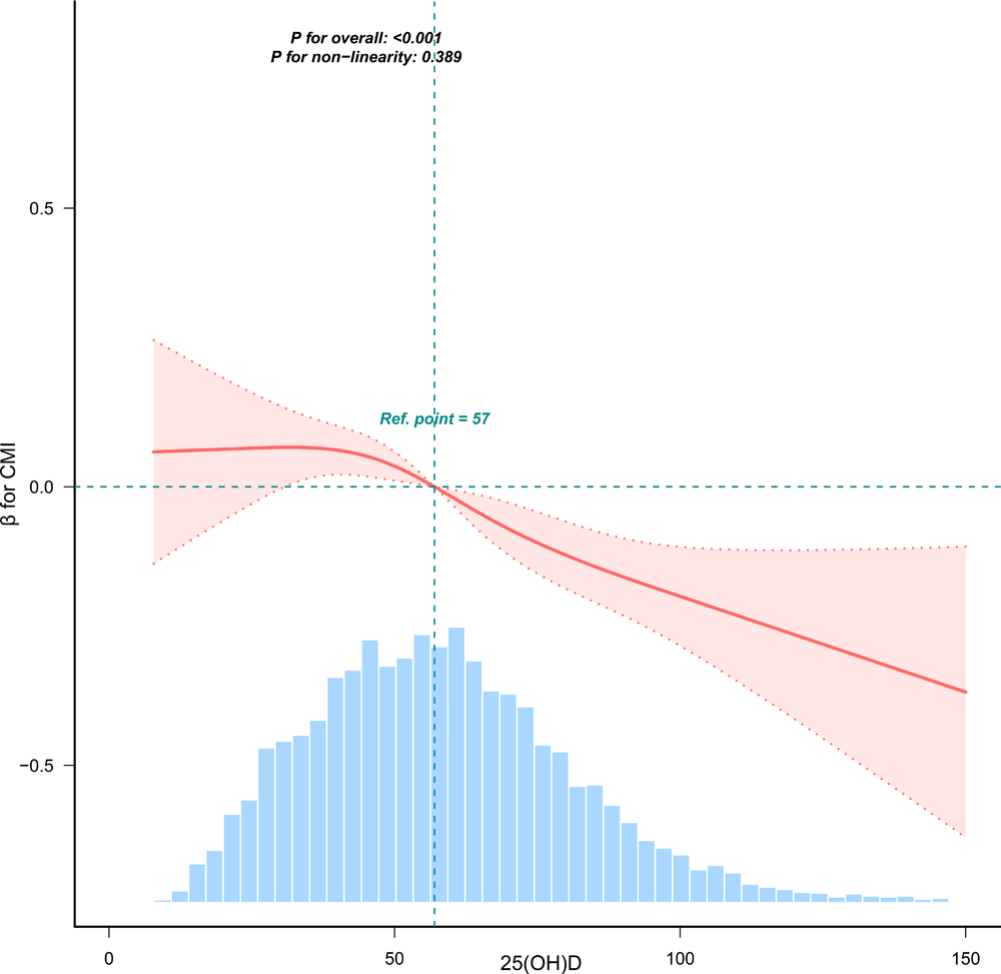
Restriction cubic spline plot of Serum 25-Hydroxyvitamin D association with Cardiometabolic Index. The light blue background histogram shows the density distribution of serum 25(OH)D levels in the study population. The solid red line represents the calculated adjusted CMI relative value, and the red shaded band represents the 95% confidence interval. The vertical dotted line indicates the reference point (serum 25(OH)D 57 units), and the regression coefficient β of this point is set to 0.

To comprehensively characterize the dose-response relationship between serum 25(OH)D and CMI, we employed 2 complementary statistical methods. First, we used a RCS model to assess for a potential nonlinear association (Fig. 2). After adjusting for all covariates, a significant overall association between serum 25(OH)D and CMI was observed (*P* for overall <.001). The curve visually demonstrates a decreasing trend in CMI as 25(OH)D levels increase. However, the test for nonlinearity was not statistically significant (P for nonlinearity = 0.389), indicating that although the curve shows a slight flattening trend in the high-concentration range, the added complexity of the spline model did not provide a significantly better fit than a simple linear model. To more specifically test for potential threshold or saturation effects, we further conducted a piecewise regression analysis (Table 4). This model identified a potential inflection point at a 25(OH)D level of 120.385 nmol/L. Below this point, 25(OH)D showed a strong negative correlation with CMI (β = −0.004, 95% CI: −0.006 to −0.002, *P* <.001); above this point, the association disappeared (β = 0.001, 95% CI: −0.010–0.013, *P* = .813). However, the likelihood ratio test, which formally compares the goodness-of-fit between the single-line and piecewise models, was not significant (*P* = .346). Taken together, the results from the RCS analysis and the threshold effect likelihood ratio test were consistent, mutually confirming that although a weakening trend was observed at very high concentrations, there is insufficient statistical evidence to support a nonlinear or threshold-based model. Therefore, within the concentration range observed in this study, the negative association between serum 25(OH)D levels and the cardiometabolic index is best described by a linear model.

**Table 4:** Threshold effect analysis of relationship of serum 25-hydroxyvitamin D with cardiometabolic index.

	Model 3	
25(OH)D	β(95%CI)	*P*-value
≤120.385	−0.004 (−0.006 to −0.002)	<.001
>120.385	0.001 (−0.01 to 0.013)	.813
Likelihood Ratio test	–	.346
Nonlinear test*1	–	.683
Nonlinear test*2	–	.383

Model 3 was adjusted for age, gender, race, poverty-income ratio, BMI, smoking, drinking, activity, sleep disorders, diabetes and hypertension.

β = coefficient, BMI = body mass index, CI = confidence interval.

### 3.4. Subgroup analysis for the association between 25(OH)D and CMI

**Figure 3.**
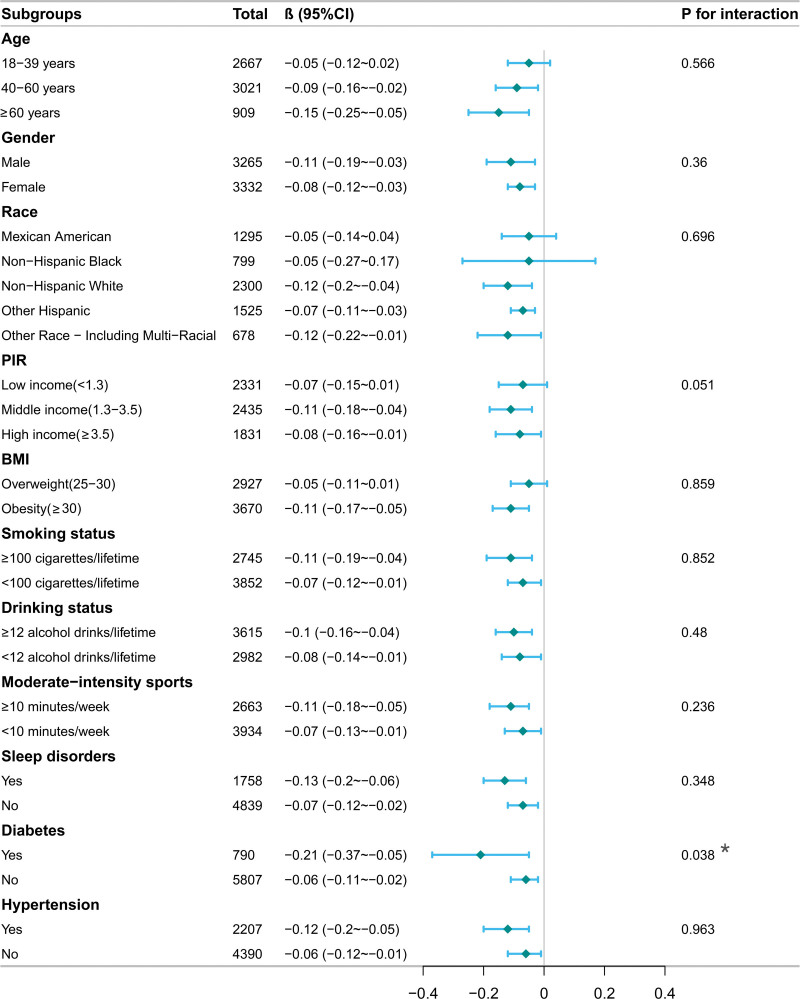
Subgroup analysis. BMI: body mass index, PIR = poverty-to-income ratio.

The results of the subgroup analysis are detailed in Figure 3. The negative association between serum 25(OH)D and CMI was largely consistent across all strata (including age, gender, race, BMI, and lifestyle factors), with most interaction *P*-values being >.05. However, diabetic status was identified as a significant effect modifier (P for interaction = 0.038). In overweight/obese adults with diabetes, serum 25(OH)D was strongly and negatively associated with CMI (β = −0.21, 95% CI: −0.37 to −0.05), an effect size substantially stronger than that in the nondiabetic population (β = −0.06, 95% CI: −0.11 to −0.02). In all other analyzed subgroups, serum 25(OH)D and CMI consistently showed a statistically significant negative association (all β values with 95% confidence intervals did not include 0).

**Figure 4.**
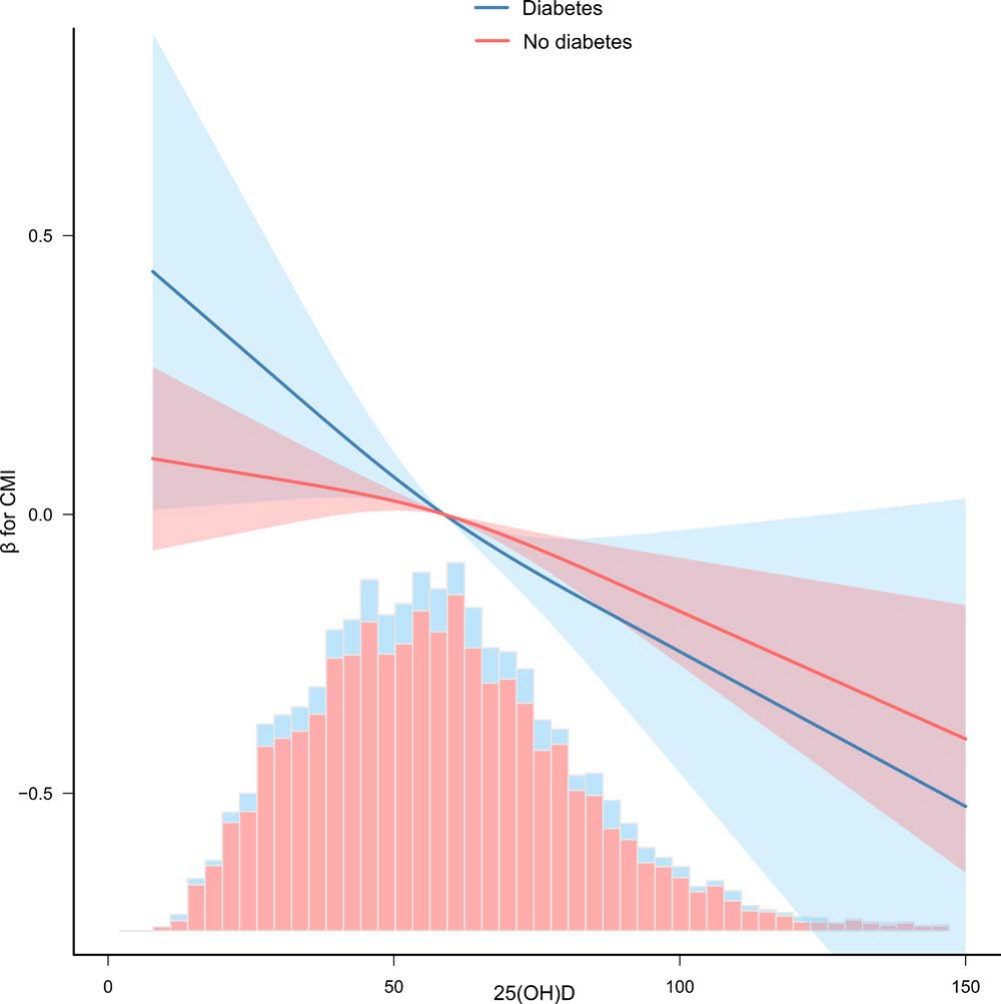
Restricted cubic spline plot of Serum 25-Hydroxyvitamin D association with cardiometabolic index stratified by diabetes status. The solid blue line and shaded area represent the regression coefficient and 95% confidence interval of diabetes population; the solid red line and shaded area represent the regression coefficient and 95% confidence interval of non diabetes population. The histogram at the bottom shows the distribution of serum 25 (OH) D levels in 2 subgroups.

In order to explore the expression of the above negative correlation in key clinical subgroups, we further evaluated the potential modification effect of diabetes status. Figure 4 intuitively shows the dose-response relationship between serum 25 (OH) D and CMI stratified by diabetes status. There is a significant difference in the shape of the 2 curves. In patients with diabetes, there is a steep, nearly linear negative correlation curve between serum 25 (OH) D level and CMI, indicating that the increase of vitamin D level is accompanied by significant improvement of cardiac metabolic health. In contrast, this negative correlation also exists in non diabetes population, but its slope is much smoother. Therefore, diabetes status is a key modifier.

### 3.5. Sensitivity analysis

To address the potential heterogeneity between overweight and obese individuals, we performed a sensitivity analysis restricted to 3670 participants with obesity (BMI ≥ 30 kg/m²). The results demonstrated that the negative association between serum 25(OH)D and CMI was not only robust but also more pronounced in this high-risk subgroup (Table 5). After full adjustment for covariates (Model 3), the association remained highly significant (β = −0.12, 95% CI: −0.18 to −0.05, *P* <.001). This finding strongly supports the robustness of our primary conclusion.

**Table 5:** Sensitivity analysis of the association between serum 25(OH)D and cardiometabolic index in obese adults.

Variable	Model 3	
	Β (95% Cl)	*P*-value
25(OH)D	−0.12 (−0.18 to − 0.05)	<.001

Model 3 was adjusted for age, gender, race, poverty-income ratio, BMI, smoking, drinking, activity, sleep disorders, diabetes and hypertension.

β = coefficient, BMI = body mass index, CI = confidence interval.

## 4. Discussion

Based on a nationally representative sample from the NHANES 2009 to 2018 database, this study systematically revealed a significant, independent, and linear negative association between serum 25(OH)D levels and the cardiometabolic index (CMI) in the high-risk population of overweight/obese adults. Critically, we discovered that this protective association is more pronounced in individuals with comorbid diabetes, suggesting that serum 25(OH)D levels may play a more pivotal role in modulating cardiometabolic health in this specific high-risk subgroup.

In our study, we employed both RCS and piecewise regression to rigorously explore the dose-response pattern. Although the RCS curve showed a flattening trend at extremely high 25(OH)D levels, neither the overall test for nonlinearity (P for nonlinearity = 0.389) nor the likelihood ratio test for a threshold model (*P* = .346) supported a nonlinear relationship. This finding challenges the conventional notion in some fields that nutrient effects are threshold-dependent and typically follow an “S”-shaped dose-response curve.^[30]^ Our data strongly suggest that in the unique pathophysiological context of obesity, the protective effect of vitamin D is continuous across a wide concentration range, with no clear “plateau” or “ceiling” effect. This may imply that for this population, which commonly experiences functional vitamin D insufficiency due to sequestration in adipose tissue, the body’s demand for and utilization of vitamin D may remain in an “unsaturated” state. Consequently, continuous supplementation is likely to yield linear health benefits. Our results are consistent with the conclusions of a large-scale observational study, which found that overweight/obese individuals may require 1.5-fold and 2 to 3-fold higher vitamin D supplementation doses, respectively, to achieve the same serum 25(OH)D levels as normal-weight individuals.^[31]^ This further highlights the necessity of focusing on and potentially prescribing higher-dose interventions for this specific population.

The negative association observed in our study may be underpinned by multiple, interconnected biological mechanisms. First, vitamin D directly intervenes in the core pathological processes of obesity: the vicious cycle of chronic low-grade inflammation and insulin resistance.^[32,33]^ In the pathophysiological milieu of obesity, expanding adipose tissue continuously releases pro-inflammatory cytokines such as TNF-α and IL-6, which are key initiators of systemic insulin resistance.^[34]^ As an effective immunomodulator, vitamin D can directly inhibit the transcription of these pro-inflammatory cytokines via its nuclear receptor (VDR), thereby mitigating the inflammatory response.^[13,35]^ Amelioration of the inflammatory environment helps preserve insulin signaling pathways, enhancing insulin sensitivity in skeletal muscle and adipose tissue.^[34]^ Improved insulin sensitivity directly leads to reduced hepatic VLDL (triglyceride-rich) synthesis and may enhance lipoprotein lipase (LPL) activity, accelerating the clearance of triglyceride-rich lipoproteins and thus lowering the TG/HDL-C ratio, directly improving CMI.^[36–38]^ Second, vitamin D also plays a direct regulatory role in insulin secretion and lipid metabolism. Pancreatic β-cells express both VDR and 1α-hydroxylase, suggesting that vitamin D may directly influence insulin secretion by modulating intracellular calcium influx.^[13]^ Furthermore, experimental studies have shown that vitamin D can upregulate genes in adipocytes involved in fatty acid oxidation and cholesterol efflux while inhibiting lipogenesis.^[39]^ This direct intervention in lipid metabolism could, in the long term, contribute to improving body fat distribution, as reflected by the waist-to-height ratio (WHtR), thereby affecting the other component of CMI. Finally, the protective effect of vitamin D is also evident in its influence on systemic regulatory systems, further strengthening its contribution to overall cardiometabolic health. Vitamin D acts as a negative regulator of the renin-angiotensin-aldosterone system (RAAS), and sufficient levels help inhibit RAAS overactivation.^[40]^ Given the critical role of the RAAS in regulating blood pressure, water-salt balance, and promoting tissue fibrosis, the indirect effects of vitamin D through this pathway may also contribute beneficially to cardiometabolic health on a more macroscopic level.^[41]^ Therefore, the action of vitamin D is not isolated to a single metabolic pathway but rather exerts a systemic, beneficial regulatory effect on the entire cardiometabolic network.

The most compelling finding of this study is the significant modifying effect of diabetic status on the association between 25(OH)D and CMI (P for interaction = 0.038). In overweight/obese individuals with comorbid diabetes, the negative association between serum 25(OH)D levels and CMI was nearly 3 times stronger (Diabetes population: β = −0.21, non diabetes population: β = −0.06) than in their nondiabetic counterparts. The coexistence of obesity and diabetes often signifies that the body is in a more severe state of chronic low-grade inflammation, oxidative stress, and insulin resistance.^[42]^ In this “high-pressure” metabolic environment, the potential protective effects of vitamin D, a known immunomodulator and anti-inflammatory hormone, may be magnified. We speculate that this “magnification” of effect does not stem from new biological pathways but rather from the existence of a larger “room for repair” in the already decompensated system of diabetes. This discovery suggests that screening for and correcting vitamin D deficiency should perhaps become a more targeted and effective intervention strategy in the comprehensive management of overweight/obese patients with diabetes. A large-scale study on a diabetic population also showed that higher 25(OH)D levels were significantly associated with reduced all-cause and cardiovascular mortality,^[43]^ which corroborates our findings and jointly highlights the necessity of monitoring vitamin D status in the management of overweight/obese patients with diabetes.

The strengths of our study include its large, nationally representative sample and the use of rigorous NHANES survey and laboratory measurement methods, which ensure the quality of the data and the external validity of the results. We comprehensively adjusted for various potential confounding factors and employed advanced statistical models (such as RCS) to explore complex dose-response relationships, and we verified the robustness of our main findings through sensitivity analyses conducted on obese individuals. Nevertheless, our study also has some limitations. First, its cross-sectional design cannot establish causality. Although we hypothesize that low vitamin D levels are a risk factor for elevated CMI, we cannot exclude the possibility of reverse causality, where a poor cardiometabolic state (potentially accompanied by less outdoor activity) leads to lower vitamin D levels. Second, a single measurement of serum 25(OH)D may not fully represent an individual’s long-term vitamin D status, which can be influenced by factors such as seasonal fluctuations.^[44]^ At the same time, although we controlled for numerous covariates, the potential for unmeasured or unknown residual confounding (such as genetic predisposition or detailed dietary patterns) still exists. Finally, our study population consisted of U.S. adults. Given the differences in genetic background, lifestyle, and dietary patterns between U.S. and other populations, such as Chinese adults, caution should be exercised when extrapolating our findings. Future studies are warranted to validate our results in diverse ethnic populations.

## 5. Conclusion

In summary, this study confirms that among overweight/obese adults in the United States, higher serum 25(OH)D levels are independently and linearly associated with better cardiometabolic health (lower CMI). This protective association is particularly prominent in patients with comorbid diabetes, suggesting its significant target value in this specific high-risk subpopulation. Future research should focus on prospective cohort studies to establish the temporal sequence of this association and ultimately, through well-designed randomized controlled trials – especially those targeting overweight/obese individuals with comorbid diabetes – to validate whether vitamin D supplementation can serve as a low-cost, high-efficacy adjunctive strategy to effectively reduce CMI and ultimately improve cardiovascular outcomes.

## Acknowledgments

We would like to thank the participants in this study.

## Author contributions

**Data curation:** Jingwen Zhang, Yuxing Tai.

**Investigation:** Sixian Wang, Mingrui Chu.

**Methodology:** Jingwen Zhang, Zhengri Cong, Qifan Guan.

**Writing – original draft:** Jingwen Zhang.

**Writing – review & editing:** Mingjun Liu, Rongsheng Jiang
